# The osteoprogenitor-specific loss of ephrinB1 results in an osteoporotic phenotype affecting the balance between bone formation and resorption

**DOI:** 10.1038/s41598-018-31190-2

**Published:** 2018-08-24

**Authors:** Agnieszka Arthur, Thao M. Nguyen, Sharon Paton, Ana Klisuric, Andrew C. W. Zannettino, Stan Gronthos

**Affiliations:** 1 Mesenchymal Stem Cell Laboratory, Adelaide Medical School, Faculty of Health and Medical Sciences, University of Adelaide, Adelaide, 5005 SA Australia; 2 South Australian Health and Medical Research Institute, Adelaide, 5000 SA Australia; 3 Myeloma Research Laboratory, Adelaide Medical School, Faculty of Health and Medical Sciences, University of Adelaide, Adelaide, 5005 SA Australia

## Abstract

The present study investigated the effects of conditional deletion of ephrinB1 in osteoprogenitor cells driven by the *Osterix* (*Osx*) promoter, on skeletal integrity in a murine model of ovariectomy-induced (OVX) osteoporosis. Histomorphometric and μCT analyses revealed that loss of ephrinB1 in sham *Osx:cre-ephrinB1*^*fl/fl*^ mice caused a reduction in trabecular bone comparable to OVX *Osx:Cre* mice, which was associated with a significant reduction in bone formation rates and decrease in osteoblast numbers. Interestingly, these observations were not exacerbated in OVX *Osx:cre-ephrinB1*^*fl/fl*^ mice. Furthermore, sham *Osx:cre-ephrinB1*^*fl/fl*^ mice displayed significantly higher osteoclast numbers and circulating degraded collagen type 1 compared to OVX *Osx:Cre* mice. Confirmation studies found that cultured monocytes expressing EphB2 formed fewer TRAP^+^ multinucleated osteoclasts and exhibited lower resorption activity in the presence of soluble ephrinB1-Fc compared to IgG control. This inhibition of osteoclast formation and function induced by ephrinB1-Fc was reversed in the presence of an EphB2 chemical inhibitor. Collectively, these observations suggest that ephrinB1, expressed by osteoprogenitors, influences bone loss during the development of osteoporosis, by regulating both osteoblast and osteoclast formation and function, leading to a loss of skeletal integrity.

## Introduction

The Eph/ephrin molecules originally identified in the hematopoietic system^1^ have subsequently been implicated in numerous developmental and pathophysiological processes^2–5^. Importantly, members of both subclasses have been associated with skeletal development and bone homeostasis, with particular focus on the signalling between EphB4/ephrinB2 and EphB2/ephrinB1 interacting pairs^6–12^. Pioneering studies in mouse proposed that bi-directional signalling between EphB4 expressing osteoblasts and ephrinB2 expressing osteoclast precursors promoted mineral formation, while inhibiting osteoclast formation and function, respectively^6,9^. Importantly, these EphB/ephrinB interactions are not limited to the communication between osteoblasts and osteoclasts, as ephrinB ligands are also expressed by osteoblasts and osteocytes^6^; and can act on neighbouring osteogenic cells, to influence osteoblast formation and function^7,10,11^. *In vitro* studies assessing human mesenchymal stem cell populations demonstrated the expression of multiple Eph/ephrin molecules, and identified EphB2/ephrinB interactions as important promoters of mineral formation^4,5,8^. Other studies have demonstrated that biomechanical loading caused an elevation in EphB2 expression in wild type mice when compared to un-loaded conditions^13,14^, with similar observations reported for ephrinB1 over-expressing transgenic mice^15^. Notably, EphB4 expression was unchanged in these studies, suggesting that in situations of active bone remodelling, mineralisation may occur through EphB2/ephrinB1 interactions independently of EphB4/ephrinB2.

Human mutations of ephrinB1 cause skeletal defects, of both the axial and appendicular skeleton, particularly coronal craniosynostosis and frontonasal dysplasia, in addition to asymmetrical lower limb shortness^16–18^. In mouse, global knockout^19,20^ of ephrinB1 or deletion within osteogenic populations, under the control of collagen type 1 promoter^21^ or Osterix promoter^12^, also result in skeletal defects similar to the human phenotype. Conversely, ephrinB1-overexpressing transgenic mice demonstrate an elevation in bone mass and strength^15^. Collectively these observations suggest that ephrinB1 has an important role skeletal formation, where the loss of ephrinB1 in osteoprogenitors during skeletal development perturbed osteoblast formation and function, and osteoclast formation^12^, by an undetermined mechanism. This observation suggests that, while ephrinB1 may be communicating with adjacent EphB expressing osteogenic cells, direct or indirect communication may occurs between osteoblasts and osteoclasts, to influence bone homeostasis. The importance of ephrinB1 in skeletal homeostasis is also evident in pathological situations. This molecule has been associated with the pathogenesis of osteosarcoma, where ephrinB1 expressed by osteosarcoma cells and blood vessels, associated with a poorer prognosis^22^. Furthermore, during lactation-induced maternal bone loss, ephrinB1 expression was upregulated, while EphB4 and ephrinB2 expression remained unaltered^23^.

Collectively, these studies suggest that ephrinB1 may play an important role in bone disease and could act as an important regulator in maintaining bone homeostasis. However, to date, little is known about the role of ephrinB1 in the context of dysregulated bone homeostasis, as occurs with osteoporosis. The present study investigated the importance of ephrinB1 expressed by the osteogenic cell lineage to maintain skeletal integrity in mature mice following ovariectomy-induced osteoporosis.

## Materials and Methods

All methods were performed in accordance with the relevant Institutional (Adelaide University) and Australian Federal Government guidelines and regulations. Animal breeding and experiments approved by the SA Pathology (BC BC01/11 and 23/11) and the University of Adelaide Animal Ethics Committee (M-2013-144). Human blood samples were isolated from normal healthy donors with informed consent, in accordance to the guidelines and regulations of the Royal Adelaide Hospital Human Ethics Committee (protocol No. 940911a).

### Animal Breeding and surgery

Animal breeding was approved by the SA Pathology (BC BC01/11) Animal Ethics Committee. All animal experiments and analyses were approved by both SA Pathology (23/11) and the University of Adelaide (M-2013-144) Animal Ethics Committees. The tTA:Osx1-GFP:Cre (hereafter referred to as *Osx:Cre*) mice^24^ were used to conditionally delete *ephrinB1* in osteogenic progenitors^25^, where *Osx:Cre* targets stromal cells, adipocytes and perivascular cells within the bone marrow. External to the skeletal system *Osx:Cre* has also been shown to target a subset of cells within the gastric and intestinal epithelium and the olfactory bulb^26,27^. To delete ephirnB1 in osteogenic progenitors, breeding was performed as previously described^12^. Briefly, 129S-Efnb1tm1Sor/J (*EfnB1*^*fl/fl*^) female mice (JAX Laboratories, Bar Harbor, Me, USA) that had been backcrossed for 10 generations to a C57BL/6 genetic background, were bred with *Osx:Cre* males to obtain *Osx:cre-EfnB1*^*fl/0*^ males. These males were bred with *EfnB1*^*fl/fl*^ females to obtain homozygote *Osx:cre-EfnB1*^*fl/fl*^ females in the osteoblast lineage (hereafter referred to as *EfnB1*_OB_^−/−^). Twelve week old C57BL/6 female *Osx:Cre* or *EfnB1*_OB_^−/−^ mice were anesthetised, there ovaries were either sited (sham control) or were ovariectomised (OVX). The animals had their dorsal skin sutured and were allowed to recover under normal housing conditions and food and water were provided *ad libitum*. Calcein (20 mg/kg) was administered by intraperitoneal injection 10 and 3 days prior to tissue collection. Samples were analysed at 12 weeks post-surgery.

### Micro-CT (μCT) scanning and analysis

Three-dimensional micro computed tomography (µCT) was performed as previously outlined of the femur^12^ and the fifth lumbar vertebrae^28^ using Micro-CT (femur -Skyscan 1076× -ray Microtomography SkyScan, Vertebrae - Skyscan 1176× -ray Microtomography SkyScan Bruker Microct, Kontich, Belgium). Briefly, femora isolated from sham and OVX mice were scanned at 9 µm resolution, Aluminium 0.5 mm filter, Excitation 5890 ms, Voltage 48 kV, Current 110 mA, rotation step 0.6 and two frame averaging. Vertebrae were scanned at 9 µm resolution, Aluminium 0.5 mm filter, Excitation 1100 ms, Voltage 75 kV, Current 196 mA, rotation step 0.8 and one frame averaging. NRecon software was used to reconstruct the femora and vertebrae, parameters were set as follows: smoothing of 1, selection of ring artefact reduction and beam-hardening of 30%, Threshold was set to 0–0.10 (femur) and 0–0.05 (vertebrae). Reconstructed bones were aligned in the same orientation using DataViewer software and then analysed using CTAn software. The femur were analysed by avoiding the primary spongiosa (0.43 mm below the growth plate), the trabecular region (1.73 mm) of the distal femora was traced as the region of interest (ROI) and analysed. The vertebral body of the fifth lumbar vertebrae was analysed as previously described^28^. Briefly, the transverse orientation was acquired, the entire vertebral body was traced within 0.3 mm below and above the end-plates of the vertebrae. The thresholding was consistent between samples. CTVol Software (SkyScan) was used to generate 3D models.

### Histology

#### Sample preparation and staining

Femora were fixed in 10% buffered formalin for 48 hours, infiltrated and embedded in methyl methacrylate (Merk Millipore, Vic, AUS). Samples were sectioned (5 μm) as previously described^9^ and mounted on gelatine coated super frost plus slides. Longitudinal sections were stained with either Toluidine blue or Tartrate Resistant Acid Phosphatase (TRAP) and counterstained with fast green, as previously described^9^. Paraffin embedded tibial 5μm sections were rehydrated and mounted in Faramount aqueous mounting media (DAKO). Collagen fibres were visualised using multiphoton and second harmonic generation imaging (Leica TCS SP8MP multiphoton microscope), with an excitation of 880 nm and emission at 440 nm, as previously described^29^. Images were processed using Fiji/Image J software^30^.

#### Histomorphometric Analysis

OsteoMeasure V3.3.02 (Osteometric, Decatur, GA) software with an Olympus DP72 Microscope (Notting Hill, Victoria, AUS) was used for histomorphometric analyses of longitudinal sections. The trabecular bone 40x magnification images below the primary spongiosa and avoiding regions in contact with the cortical bone were analysed, in a 0.72 mm^2^ region for Calcein, and 0.96 mm^2^ region for Toluidine Blue and TRAP^+^ osteoclasts analysis. Bone formation rate was determined by measuring the distance between the two mineralisation fronts that were labelled with Calcein and analysed with OsteoMeasure software.

#### Flow cytometric analysis

Flow cytometric analysis of osteogenic populations was performed as previously described^31–33^. Briefly, compact bone was obtained by crushing the femora and tibiae, washed with PBS + 2%FCS, and then crushed fractions were cut into small fragments and enzymatically digested with collagenase type I (400 U/mL) and DNase I (50 U/mL) for 45 min at 37 °C, while shaking. Cells were washed, filtered through a 70 μm strainer and centrifuged for 10 min at 800 *g*. Resuspended cells together with flushed bone marrow were incubated in mouse gamma globulin, washed and stained with biotinylated lineage-specific antibodies. Flow cytometric analysis was performed on BD LSR Fortessa X20 (BD Biosciences). The frequency of osteoblasts (Lin^−^CD45^−^CD31^−^Sca-1^−^CD51^+^), and osteoprogenitor (Lin^−^CD45^−^CD31^−^Sca-1^+^CD51^+^) was determined using FlowJo software (University of Washington, WA) as previously described^(26–28)^.

#### Analysis of bone turnover serum markers

Mice were anesthetised and blood serum samples collected following cardiac puncture. The levels of circulating CTX-1 was analysed using RatLaps CTX-I EIA ELISA (AC-06F1; ids) as recommended by the manufacturer (Immunodiagnostic Systems, Boldon Business Park, UK).

#### RNA isolation

RNA isolation, cDNA synthesis and real time PCR were conducted as previously described^32^, using β-actin and human-specific B subclass Eph primers^4,5^; and Cathepsin K (NC_018912.2 Fwd: 5′-GGCCAACTCAAGAAGAAAACTG-3′, Rev: 5′-TCTCTGTACCCTCTGCATTTAGC-3′); c-fms (NM_005211 Fwd: 5′-AGCTTGGCATGGTCAGGGAA-3′, Rev: 5′-GAAGGTAGCGTTGTTGGTGC-3′); RANK (NM_003839 Fwd: 5′-GCTGTAACAAATGTGAACCAGGA-3′, Rev: 5′-GCCTTGCCTGTATCACAAACT-3′); CXCR4 (NM_003467 Fwd: 5′-CAGCAGGTAGCAAAGTGACG-3′, Rev: 5′-GTAGATGGTGGGCAGGAAGA-3′); ephrinB1 (Fwd 5′ AACAAGCCACACCAGGAAAT 3′, Rev 5′ GCTCCCATTGGACGTTGAT 3′).

#### Osteoclast differentiation and resorption assays

Osteoclast Differentiation *In Vitro*: Human peripheral blood mononuclear cells (hPBMNC) were isolated from buffy coats from normal healthy donors. All experiments were performed with informed consent from each donor, in accordance with relevant guidelines and regulations of the Royal Adelaide Hospital Human Ethics Committee (protocol No. 940911a) as previously described^34^. The cells were seeded (0.5 × 10^6^ per 0.32 cm^2^) and cultured overnight in osteoclast basal media (Minimum Essential Medium Alpha Modified (Sigma), 10% foetal calf serum (FCS), 1% penicillin streptomycin, 1% l-glutamine) and 25 ng/ml macrophage colony stimulating factor (M-CSF) (Miltenyi Biotec, NSW, AUS) (Day -1) in 96-well plates (TRAP staining and viability analysis) or Corning Osteo Assay Surface Muliple Well plates (Sigma) (resorption analysis). The following day, the Human IgG-Fc or ephrinB1-Fc at 1 µg/ml (R&D systems) were pre-clustered with biotinylated anti-goat IgG (Jackson ImmunoReseaarch, West Grove, PA, 2.4 mg/ml) in osteoclast media (osteoclast base media supplemented with 25 ng/ml M-CSF, 10^−8^M dexamethasone (Hosporia Aust. Mulgave, Vic, Aust.), and 10^−8^M 1α,25-dihydroxyvitaminD3 (Cerilliant Corporation, TX) for 1 hour at room temperature prior to being added to osteoclast cultures. Media was changed three-times per week. From day 7, osteoclast media was also supplemented with 50 ng/ml RANKL (Miltenyi Biotec). On day 14, wells were processed for either RNA isolation or TRAP staining using the Sigma Acid Phosphate, Leukocyte (TRAP) kit (Sigma).

Bone marrow (BM) cells flushed from the femur and tibia of mice were cultured overnight, then non-adherent cells were collected and seeded (3.1 × 10^5^ cells/cm^2^) in α-MEM supplemented with 75 ng/ml of M-CSF (GF053; Merck Millipore) and RANKL (GF091; Merck Millipore). Medium was replaced every 3 days. On day 6, RNA was isolated, cDNA synthesised, and qPCR performed.

EphB2 Inhibitor Assays: Human hPBMNC were incubated for 1 hour in half the final volume of osteoclast media (outlined above) containing 100 µM blocking peptide specific for EphB2 (SNEWILPRLPQH) or control peptide (RTVAHHGGLYHTNAEVK) (Mimotopes, Vic, Aust.). During this 1-hour incubation 2 µg/ml of ephrinB1-Fc (final concentration, 1 µg/ml) was clustered (outlined above) in the other half of the final volume of osteoclast media. The remaining 100 µM blocking peptide (SNEW or RTVA) was then combined with the clustered ephrinB1-Fc. This ephrinB1-Fc-blocking peptide osteoclast media was immediately added to hPBMNC that had been incubated with the blocking peptide. Media changes with ephrinB1-Fc and blocking peptides were carried out as outlined above.

Cell Viability Assays: On day 3 and 7 following osteoclast induction, viability and metabolism were assessed by WST-1 (Sigma). Briefly, WST-1 was diluted in Pheno-Red free alpha-modification media (Sigma) containing 25 ng/ml M-CSF. Cells and control blank wells were incubated with the diluted WST-1 for 1 hour at 37 °C, after which time the absorbance was read at 450 nm. Experimental values were normalised to the blank. On day 21, cells in the Corning Osteo Assay Surface Multiple Well plates were lysed with 6% sodium hypochlorite, washed with water three times and then left to dry. Whole wells were imaged and analysed with Image J software.

#### Osteogenic Differentiation *In Vitro*

Bone derived mesenchymal stromal cells isolated from murine tibia and femora were cultured in αMEM supplemented with 20% (v/v) FCS, 2 mM l-glutamine, 1 mM sodium pyruvate, 10 mM HEPES buffer, 50 U/ml penicillin, 50 μg/ml streptomycin, 100 µM l-ascorbate-2-phosphate (Wako Pure Chemical Industries, Richmond, VA, USA), 10 nM dexamethasone (RAH688A; Royal Adelaide Hospital, Adelaide, SA, Australia), and 4 mM KH2PO4 (Asia Pacific Specialty Chemicals Limited, Seven Hills, NSW, Australia). Cultures were stained with Alizarin red (A5533-25G; Sigma-Aldrich) to identify mineral deposits. Extracellular calcium (Ca2^+^) was measured by Calcium Arsenazo III (TR29226; Thermo Fisher Scientific) and normalized to DNA per well with a PicoGreen dsNDNA quantitation kit (P11496; Thermo Fisher Scientific). This analysis was conducted in triplicate for each mouse.

#### Statistical analysis

Microsoft GraphPad Prism 5 (La Jolla, CA) was used for data and statistical analysis. Student t-test, one-way ANOVA with multiple comparisons and two-way ANOVA, using a Tukey’s multiple comparisons post-hoc test was used throughout the study as specified. Statistical significance is represented as p ≤ 0.05.

## Results

### Confirmation of osteogenic-specific deletion of ephrinB1 in EfnB1_OB_^−/−^ mice

It has previously been reported that the deletion of ephrinB1 under the control of the *Osterix* promoter impairs endochondral ossification, compromising skeletal development and integrity^12^. The study demonstrated that ephrinB1 gene and protein levels were significantly reduced in a heterogeneous population of cultured osteogenic cells isolated from *Osx:Cre* and *EfnB1*_OB_^−/−^ mice^12^. The present study confirmed that following osteogenic differentiation of osteogenic cells isolated from *Osx:Cre* and *EfnB1*_OB_^−/−^ mice, there was a significant reduction in the formation of mineral nodules and extracellular calcium levels in osteogenic cells isolated from *EfnB1*_OB_^−/−^mice when compared to *Osx:Cre* controls (Supplementary Fig. 1A,B). This observation correlated with a significant reduction in *ephrinB1* gene expression in stromal cells isolated from *EfnB1*_OB_^−/−^ mice following osteogenic induction when compared to the *Osx:Cre* control (Supplementary Fig. 1C). Furthermore, to demonstrate that the *Osterix-Cre* promoter was active specifically within the stromal population within the skeleton, marrow cells flushed from the long bones were placed under osteoclastic differentiation conditions. Following 6 days in osteoclast inductive conditions, ephrinB1 gene expression levels were found to be comparable in osteoclasts/monocyte populations isolated from *Osx:Cre* control and *EfnB1*_OB_^−/−^ mice (Supplementary Fig. 1D–F). These observations confirmed that ephrinB1 under the control of the *Osterix-Cre* promotor was regulated in the osteogenic cell lineage and not the haematopoietic population within the bone marrow.

### The loss of ephrinB1 in osteoprogenitors is sufficient to induce an osteoporotic bone phenotype

**Figure 1:**
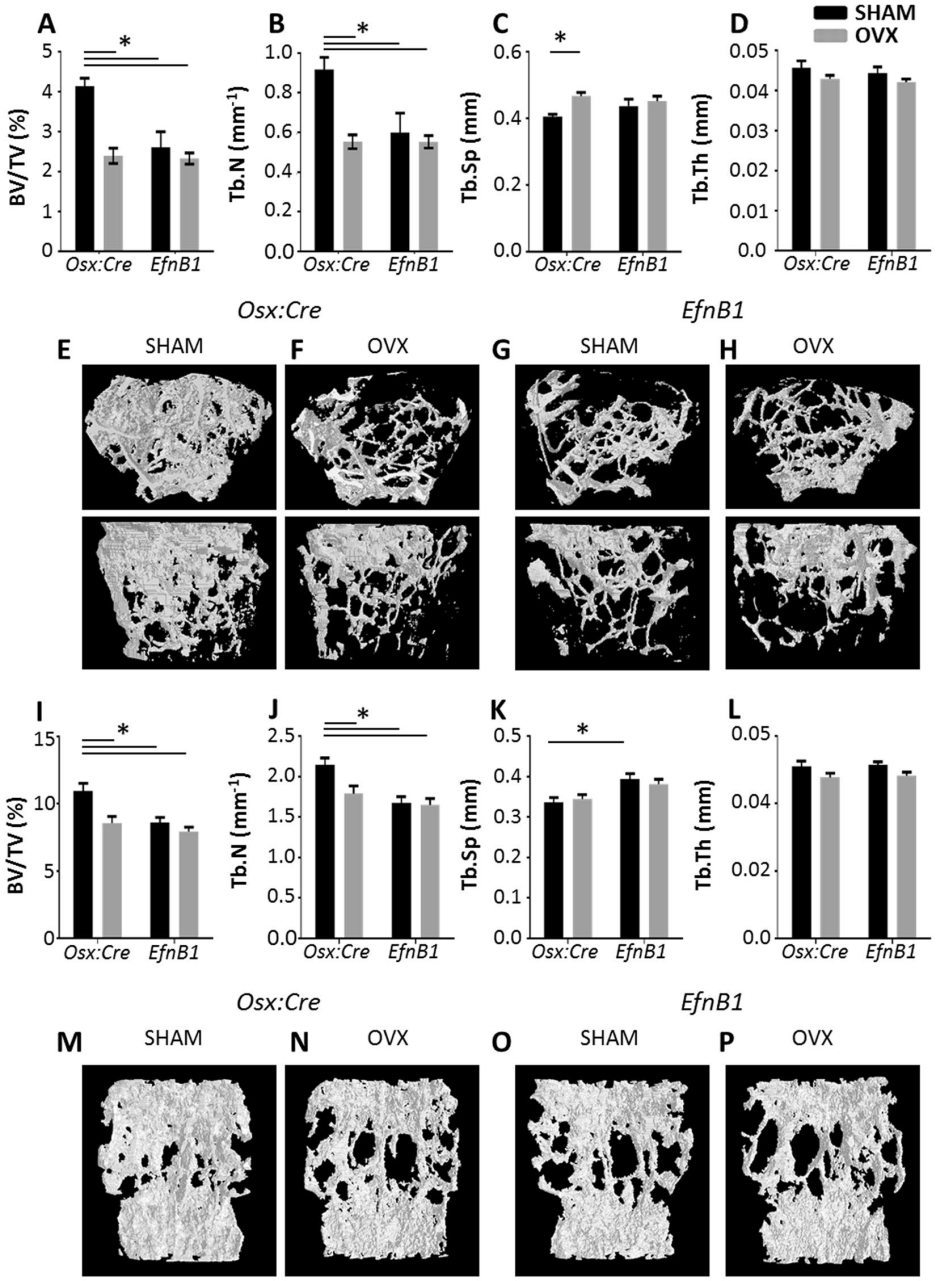
The loss of EfnB1 in osteoprogenitors alone induces an osteoporotic bone phenotype. (**A–P**) µCT analysis of the trabecular region of the (**A–D**) distal femoral and (**I–L**) fifth lumbar vertebral body of *Osx:Cre* (*Osx*) and *EfnB1*_OB_^−/−^ (*EfnB1*) mice that had undergone sham or ovariectomy (OVX) surgery; represented as (**A,I**) Bone to tissue volume (BV/TV), (**B,J**) trabecular number (Tb.N), (**C,K**) trabecular separation (Tb.Sp) and (**D,L**) trabecular thickness (Tb.Th). (**E–H,M–P**) Representative images of 3D µCT from the (**E–H**) femur (in cross-sectional (top) and longitudinal (bottom) orientation) and (**M–P**) vertebral body of (**E,G,M,O**) sham and (**F**,**H,N,P**) ovariectomised (OVX) mice. All statistical analysis performed by 2-way ANOVA, multiple comparisons, n = 6–9 mice/condition/strain, *p < 0.05.

**Figure 2:**
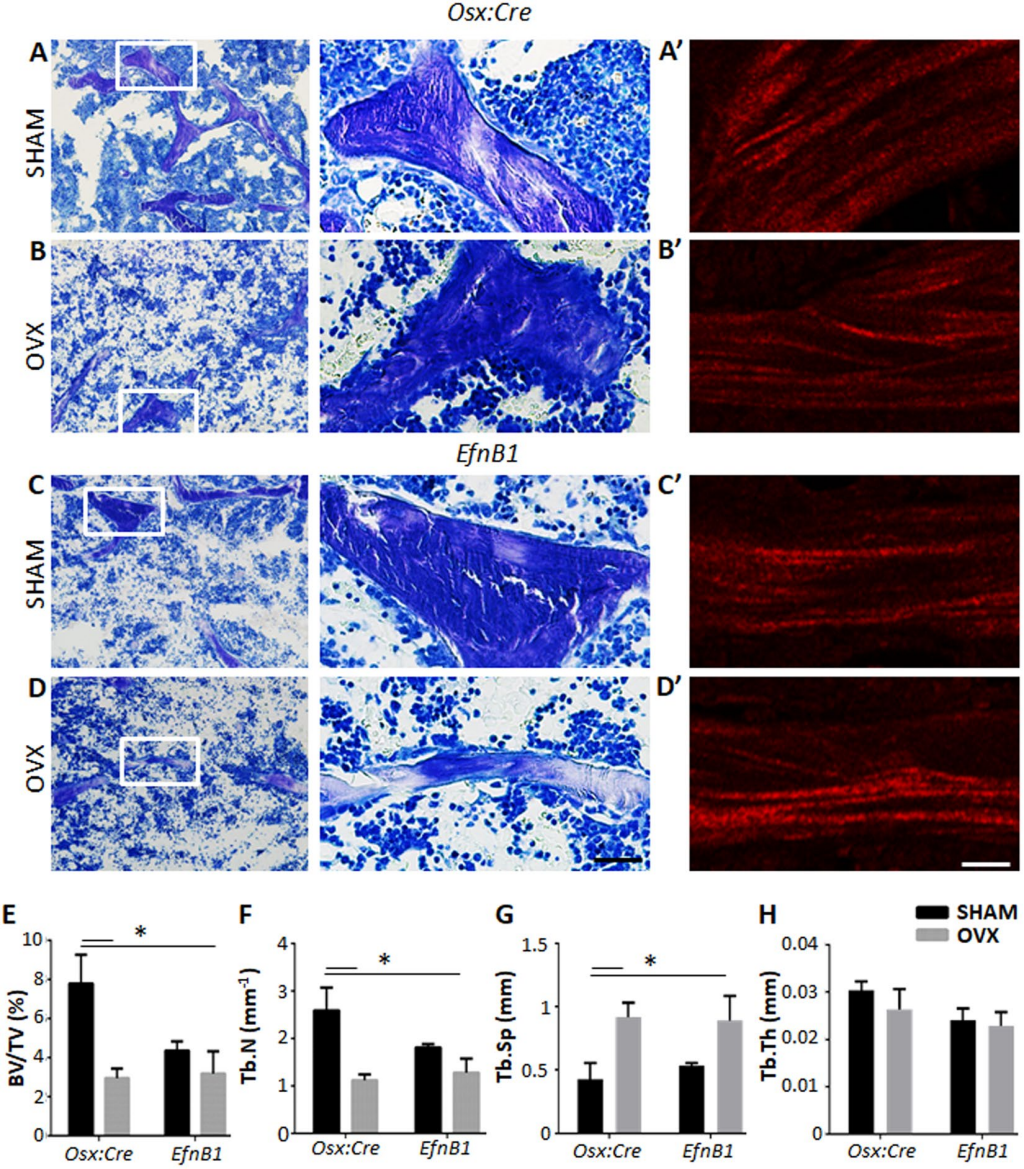
The loss of EfnB1 by osteoprogenitors alone impairs trabecular bone architecture similar to ovariectomised mice. (**A–D**) Representative toluidine blue stained sections of (**A,B**) Osx:Cre (Osx) control and (**C,D**) EfnB1_OB_^−/−^ (EfnB1) femora, 12 weeks following (**A–C**) sham and (**B–D**) ovariectomy (OVX) surgery. (**A’**–**D’**) Representative images of collagen type I fibres. (**E–H**) Histomorphometric analysis of the trabecular region of the distal femora post-surgery represented as (**E**) percentage of bone to tissue volume, (**F**) trabecular number, (**G**) trabecular separation, and (**H**) trabecular thickness. All statistical analysis performed by 2-way ANOVA, multiple comparisons, *p < 0.05, n = 3–5 samples/strain/condition. Magnification A-D and A’–D’ = 10 µM.

We investigated whether ephrinB1, expressed by cells of the osteogenic lineage contributed to bone remodelling using a model of ovariectomy (OVX)-induced osteoporosis. Ovariectomy or sham surgery was performed on female homozygote *EfnB1*_OB_^−/−^ mice, or *Osx:Cre* control mice and skeletal parameters were assessed at 12 weeks post-surgery. Three dimensional µCT analysis revealed that OVX induced an osteoporotic phenotype, as evidenced by a significant reduction in bone to tissue volume and trabecular number in both the femora (Fig. 1A–F), and vertebra (Fig. 1I–N) in OVX *Osx:Cre* mice compared to sham *Osx:Cre*. Interestingly, the loss of ephrinB1 in sham *EfnB1*_OB_^−/−^ mice resulted in a skeletal phenotype comparable to the OVX *Osx:Cre* mice, which was not further exacerbated following OVX in *EfnB1*_OB_^−/−^ mice, that was observed in both the femora and the vertebra (Fig. 1). A significant increase in trabecular separation was observed in the femora of OVX *Osx:Cre* mice compared to sham *Osx:Cre* (Fig. 1C), however, this was not maintained in the vertebrae (Fig. 1K). Notably, a significant increase in trabecular separation within the vertebrae was observed between sham *Osx:Cre* and sham *EfnB1*_OB_^−/−^ mice, while OVX *EfnB1*_OB_^−/−^ mice did not reach statistical significance. Furthermore, there was no significant difference observed in the trabecular thickness between any of the cohorts (Fig. 1D,L). Confirmatory 2D histomorphometric analysis of toluidine blue stained sections of trabecular regions within the distal femora, showed a significant reduction in bone to tissue volume, trabecular number and significant increase trabecular separation of OVX-induced *Osx:Cre* control mice when compared to sham-treated *Osx:Cre* control mice. Consistent with the µCT analysis, there was no significant difference in trabecular thickness between any of the groups. Furthermore, homozygote sham and OVX *EfnB1*_OB_^−/−^ mice displayed a similar phenotype to OVX-induced *Osx:Cre* mice (Fig. 2). Multiphoton and second harmonic generation imaging demonstrated that the perturbation in trabecular architecture was not due to an abnormal pattern of collagen type I deposition (Fig. 2A’–D’). Taken together, these observations suggest that ephrinB1 deletion in osteogenic cells can recapitulate an osteoporotic phenotype that cannot be further exacerbated by the oestrogen deprivation that accompanies OVX.

### Osteoblast maturation and function is compromised by the loss of ephrinB1

**Figure 3:**
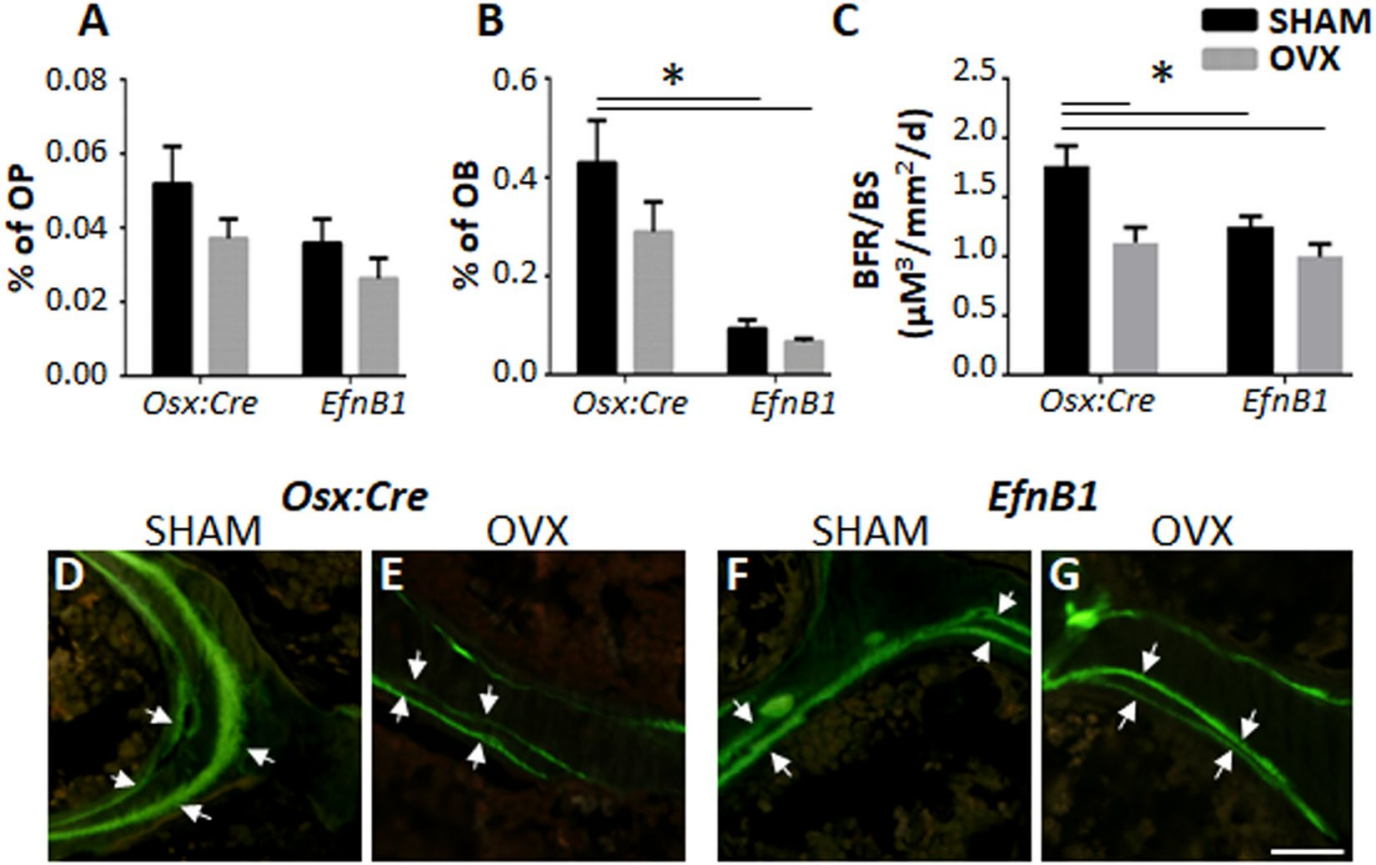
The loss of EfnB1 by osteoprogenitors inhibits osteoblast maturation and bone formation rate. (**A,B**) Fluorescent cytometric analysis of the percentage of (**A**) osteoprogenitors (**OP**, Sca-1^+^/Lin^−^/CD31^−^/CD45^−^/CD51^+^), and (**B**) osteoblasts (**OB**, Sca-1^−^/Lin^−^/CD31^−^/CD45^−^/CD51^+^), isolated from flushed and digested femora and tibia of *Osx:Cre* and *EfnB1*_OB_^−/−^ sham and ovariectomised (OVX) mice 12 weeks post-surgery (n = 5–6 samples/strain/condition). (**C**) Bone formation rate (BFR) was analysed by Calcein labelling (n = 3–5 samples/strain/condition) (**D–G**) Representative double labelled Calcein images. All statistical analysis performed by 2-way ANOVA, multiple comparisons, *p ≤ 0.05. Magnification D–G = 5 µM.

Flow cytometric analysis was performed as previously described^31–33^, to enumerate the incidence of cells of the osteogenic lineage in the femora and tibiae of sham *EfnB1*_OB_^−/−^ homozygote females and sham and OVX *Osx:Cre* mice (Fig. 3A–C). While there was a reduction in the osteogenic progenitors (Sca-1^+^/Lin^−^/CD31^−^/CD45^−^/CD51^+^) population between *Osx:Cre* and *EfnB1*_OB_^−/−^ samples, this did not reach significance (Fig. 3A). However, there was a significant reduction in the proportion of osteoblasts (Sca-1^−^/Lin^−^/CD31^−^/CD45^−^/CD51^+^) between sham *Osx:Cre* mice and both sham and OVX *EfnB1*_OB_^−/−^ mice (Fig. 3B). These findings were consistent with an observed reduction in the bone formation rate of OVX *Osx:Cre* mice, compared to sham *Osx:Cre* mice, and compared to both sham and OVX *EfnB1*_OB_^−/−^ mice (Fig. 3C–G). These observations suggest that the cell-autonomous loss of ephrinB1 in osteogenic progenitors inhibits osteogenic maturation and function.

### Osteoclast formation and function is affected by EphB2 forward signalling

**Figure 4:**
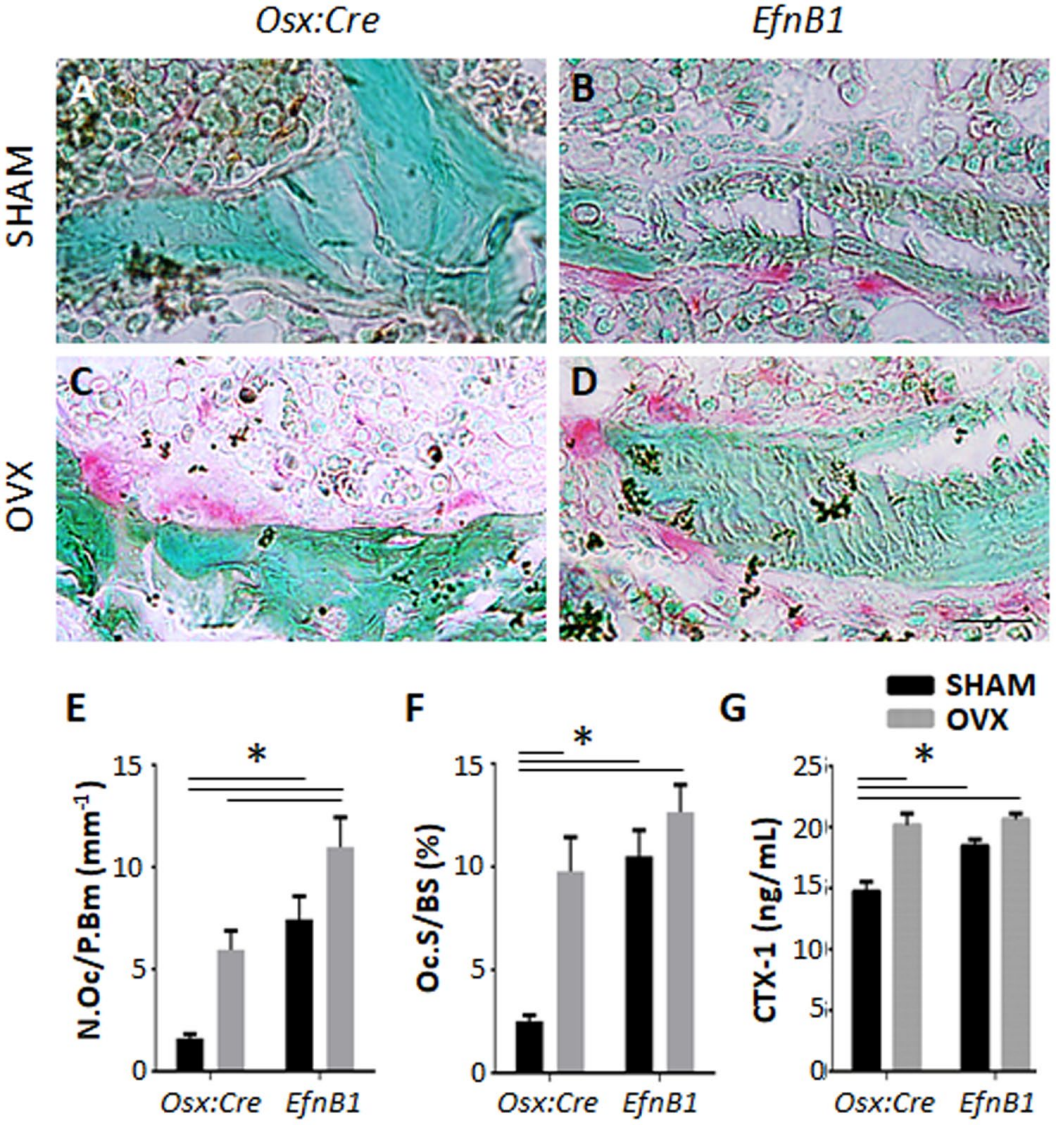
The loss of EfnB1 by osteoprogenitors influences osteoclast formation and function. (**A–D**) Representative methacrylate embedded sections of the distal femora from (**A,B**) sham and (**C,D**) ovariectomised (OVX) (**A,C**) *Osx:Cre* (*Osx*) control and (**B,D**) *EfnB1*_OB_^−/−^ (*EfnB1*) mice 12 weeks post-surgery stained with TRAP. (**E,F**) Histomorphometric analysis quantitating (**E**) the number of TRAP^+^ osteoclasts lining the bone perimeter, and (**F**) the percentage of osteoclast surface to bone surface within the trabecular region of the distal femora of *Osx:Cre* and *EfnB1*_OB_^−/−^ sham and OVX mice (n = 4–5 samples/strain/condition). (**G**) Circulating collagen type I was measured by ELISA (n = 5–8 samples/strain/condition). All statistical analysis performed by 2-way ANOVA, multiple comparisons, *p ≤ 0.05. Magnification A–D = 10 µM.

To determine if osteoclast formation and activity is EphB2 dependent, and therefore affected in response to the loss of ephrinB1 by osteoprogenitors and mature osteogenic populations, methacrylate embedded sections were stained with TRAP, and the number of TRAP + multinucleated osteoclasts were enumerated within the trabecular region of the distal femora of sham and OVX mice (Fig. 4A–F). Twelve weeks post-surgery, OVX *Osx:Cre* mice presented with significantly greater numbers of TRAP^+^ osteoclasts per bone perimeter compared to sham *Osx:Cre* mice (Fig. 4E). Similarly, sham *EfnB1*_OB_^−/−^ mice displayed comparable numbers of TRAP + osteoclasts to OVX *Osx:Cre* mice, while OVX *EfnB1*_OB_^−/−^ mice displayed significantly more TRAP + osteoclasts when compared to OVX *Osx:Cre* mice (Fig. 4E). The proportion of osteoclast surface to bone surface was significantly greater in OVX *Osx:Cre* mice compared to sham *Osx:Cre* mice. However, the osteoclast surface to bone surface was comparable between sham and OVX *EfnB1*_OB_^−/−^ mice; and OVX *Osx:Cre* mice (Fig. 4F). These data suggest that the loss of ephrinB1 within osteoprogenitor and mature osteogenic populations influences osteoclast formation. To determine if osteoclast function was also altered due to the loss of ephrinB1, serum samples were used to investigate circulating levels of degraded collagen type 1. A significant increase in circulating degraded collagen type 1 (CTX-1) was identified in OVX *Osx:Cre* mice, and both sham and OVX *EfnB1*_OB_^−/−^ mice, when compared to sham *Osx:Cre* control mice (Fig. 4G). Importantly, *ephrinB1* gene expression was similar between *EfnB1*_OB_^−/−^ and *Osx:Cre* mice (Supplementary Fig. 1D). These findings suggest that ephrinB1 expressed by the osteogenic population affects osteoclast formation and function rather than in a cell-autonomous manner.

**Figure 5:**
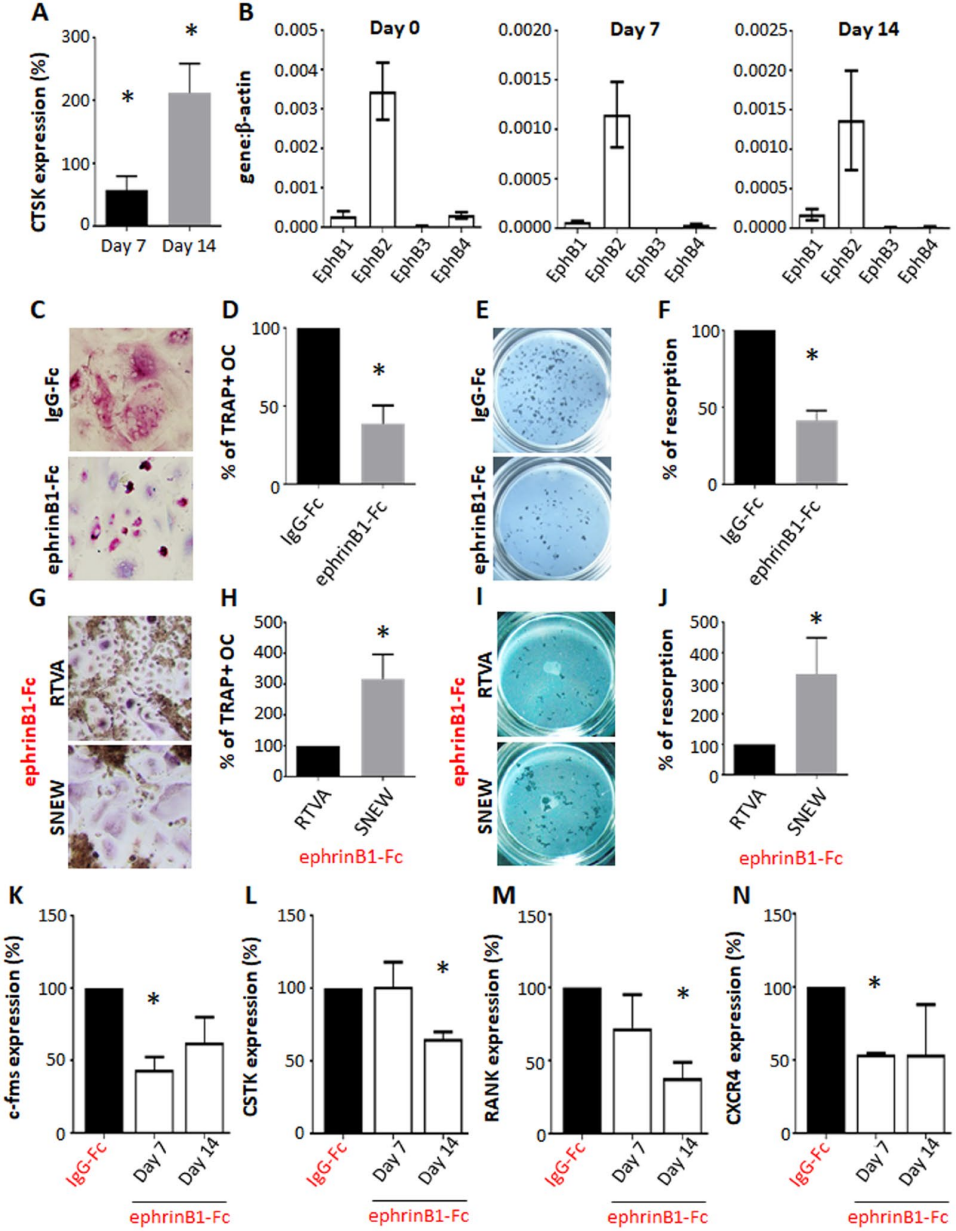
Mechanism of osteoclast function in human *in vitro* osteoclast differentiation assays. (**A**) Gene expression of Cathepsin K (CTSK) expressed as a fold increase compared to day 0 (n = 9 human donors, *p < 0.05, one-way ANOVA, multiple comparisons). (**B**) Expression profile of EphB receptors identified on human PBMNC prior to (Day 0) and under osteoclastogenic conditions (Day 7 and 14). (**C–F**) Human PBMNC placed under osteoclastic conditions in the presence of human IgG-Fc control or ephrinB1-Fc for 14 days were (**C**) stained for TRAP^+^ osteoclasts, (**D**) enumerated and represented as a percentage of TRAP^+^ multi-nucleated osteoclasts (OC) relative to the IgG-Fc control (n = 6 independent donors, *p < 0.05, Student t-test). (**E,F**) Samples were cultured for 21 days on Corning Osteo Assay Surface Muliple Well plates and (**E**) imaged. (**F**) The proportion of resorbed surface area was quantitated and represented as a percentage relative to the human IgG-Fc control (n = 3 human donors, *p < 0.05, Student t-test). (**G–J**) Samples cultured under osteoclastic conditions with ephrinB1-Fc in the presence of either the scramble peptide control (RTVA) or the EphB2-specific blocking peptide (SNEW). (**G–H**) On Day 14 samples were (**G**) stained for TRAP^+^ osteoclasts, (**H**) quantitated and represented as a percentage of TRAP^+^ OC relative to RTVA (n = 3 independent human donors, *p < 0.05, Student t-test). Resorptive function was assessed on day 21 of culture (**I–J**). Corning Osteo Assay plates were (**I**) imaged, (**J**) quantitated and represented as a percentage of resorption capacity relative to RTVA (n = 3 independent human donors, *p < 0.05, Student t-test). (**K–N**) Gene expression of (**K**) c-fms (**L**) Cathepsin K (CSTK), (**M**) RANK and (**N**) CXCR4 were analysed 7 or 14 days following osteoclast induction and represented as a percentage of gene expression when cultured in the presence of ephrinB1-Fc relative to human IgG (n = 2 human donors, *p < 0.05, Student t-test).

*In vitro* osteoclastogenesis studies were conducted to determine the direct role of ephrinB1 during human osteoclast formation. Gene expression analysis confirmed osteoclast differentiation of Human peripheral blood mononuclear cells (hPBMNC) with increasing expression levels of CathepsinK during osteoclastogenesis (Fig. 5A). Moreover, profiling of the Eph receptors identified that the cognate ephrinB1 receptor, EphB2, was the most highly expressed EphB receptor identified on hPBMNC during osteoclast differentiation (Fig. 5B). As the mouse studies demonstrated that loss of ephrinB1 by osteoprogenitors enhanced osteoclast formation, functional studies were conducted to confirm whether stimulation of hPBMNC with ephrinB1 alone, could inhibit osteoclast differentiation. Human PBMNC were cultured under osteoclastogenic conditions in the presence of either soluble ephrinB1-Fc or the human IgG control. The data showed that ephrinB1-Fc significantly inhibited TRAP^+^ multinucleated osteoclast formation (Fig. 5C,D). Consistent with these findings, osteoclast bone resorption activity was significantly inhibited, in the presence of soluble ephrinB1-Fc (Fig. 5E,F). Importantly, the presence of ephrinB1-Fc did not influence cell viability or metabolism during osteoclast formation, as demonstrated by no change in amount of formazan dye formed following incubation with tetrazolium salt, WST-1 (Supplementary Fig. 2).

To confirm that ephrinB1 was blocking osteoclast formation and function through the EphB2 receptor, inhibitor studies were conducted utilising the EphB2 specific blocking peptide (SNEW). SNEW specifically binds to the ligand binding cleft of EphB2, competing with ephrin ligand binding^35^. Human PBMNC cultured under osteoclastogenic conditions were incubated with SNEW or a scrambled peptide control (RTVA) prior to culturing with ephrinB1-Fc. The competitive binding of SNEW to EphB2 in the presence of ephrinB1 significantly ameliorated TRAP^+^ osteoclast formation (Fig. 5G,H) and resorption (Fig. 5I,J) when compared to the scrambled control peptide (RTVA). This observation confirms that ephrinB1 interaction with EphB2 expressing osteoclast precursors inhibits osteoclast formation and subsequently function. Furthermore, supportive gene expression studies showed that transcript levels of osteoclast associated factors, Cathepsin K, c-fms, RANK and CXCR4 were significantly decreased during osteoclastogenesis in the presence of soluble ephrinB1 (Fig. 5K–N). These observations suggest that ephrinB1 binding, through the EphB2 receptor, inhibits osteoclast formation.

## Discussion

In the present study, targeted deletion of ephrinB1, under the control of the osterix promoter, induced an osteoporotic phenotype in sham treated *EfnB1*_OB_^−/−^ mice, which was comparable to that observed in OVX *Osx:cre* control mice. Notably, the osteoporotic phenotype was not exacerbated in *EfnB1*_OB_^−/−^ mice following OVX. We observed a significant reduction in trabecular bone between OVX *Osx:Cre* mice compared to sham *Osx:Cre* controls. The loss of *ephrinB1* in sham operated *EfnB1*_OB_^−/−^ mice resulted in a similar trabecular architecture to OVX induced *Osx:Cre* and *EfnB1*_OB_^−/−^ mice, as demonstrated by μCT analysis of two independent sites, the femur and the fifth lumbar vertebrae, and histomorphometric analyses of the femur. The formation of type I collagen fibres is essential for skeletal architecture and integrity, where interactions of Eph family members with the extracellular matrix can influence extracellular matrix organisation^36,37^. However, the present study failed to observe any noticeable differences in the patterns of collagen deposition between *Osx:Cre* controls and *EfnB1*_OB_^−/−^ mice. Additionally, measurement of oestrogen levels in serum failed to detect any changes in oestrogen levels (data not shown) to explain the difference observed in skeletal architecture between sham *EfnB1*_OB_^−/−^ mice. However, the reduction in trabecular bone observed between OVX *Osx:Cre* mice, OVX *EfnB1*_OB_^−/−^ mice and sham *EfnB1*_OB_^−/−^ mice was associated with a significant reduction in bone formation rate within the femur. As the *Osx:Cre* mouse line has been reported to exhibit changes in skeletal parameters, including cortical bone augmentation and accrual^38^, the present study utilized *Osx:Cre* mice rather than *EfnB1*^*fl/fl*^ mice as the control. Therefore, the osteoporotic-like phenotype observed in sham *EfnB1*_OB_^−/−^ mice did not appear to be a consequence of the *Osx:Cre* background, but rather due to deletion of ephrinB1 in osteogenic cells, which could not be exacerbated following OVX.

The importance of ephrinB1 in skeletal formation can be observed in individuals who display cranial and skeletal defects and harbour functional mutations in the ephrinB1 gene^16–18^. Similar skeletal abnormalities have been reported in global and targeted deletion of ephrinB1 during murine development^12,19–21^. At the molecular level, ephrinB1 mediates mineral formation by activating *Osterix* expression^21^. EphB2 activation of ephrinB1 reverse signalling results in the dephosphorylation of TAZ from its complex with the PDZ domain of ephrinB1, PTPN13 and NHERF1. The dephosphorylated TAZ translocates to the nucleus and induces osterix expression, osteogenic differentiation and subsequently mineral production^21^. Consistent with these observations, human studies have shown that ephrinB1 is the most highly expressed EphB ligand on human BMSC, and activation of ephrinB1 promotes BMSC osteo/chondrogenic differentiation *in vitro*^5^. Flow cytometric analysis found a trend of lower numbers of osteoprogenitors but significantly reduced osteoblast numbers in both the sham and OVX treated *EfnB1*_OB_^−/−^ mice, compared to sham *Osx:cre* control mice. Given the reported redundancy between EphB/ ephrinB family members, where ephrinB2/ EphB4 signaling within the osteogenic lineage has previously been shown to be important in the late stages of osteoblast differentiation^10^. Mice lacking ephrinB2 in osteoblasts exhibit delayed bone mineralization, significant reduction in bone stiffness, and a reduction in osteoblast differentiation in the presence of parathyroid hormone^39^. Therefore, compensation by ephrinB2 reverse signalling in mature bone cells may be a contributing factor in preventing an exacerbated osteoporotic phenotype in OVX *EfnB1*_OB_^−/−^ mice as has been suggested in other systems^6,40,41^. However, this is hypothesis requires furthermore investigation.

In the present study, both sham and OXV induced *EfnB1*_OB_^−/−^ mice displayed higher osteoclast numbers compared to *Osx:cre* OVX-induced mice. These data are consistent with previous studies demonstrating that the loss of ephrinB1 by osteoprogenitors stimulated an increase in the number of osteoclasts during skeletal development^12^. The findings presented here suggest that ephrinB1 activation may act as a contact-dependent regulator of osteoclast differentiation. Importantly, histomorphometric analysis of the distal femur identified that OVX-induced *EfnB1*_OB_^−/−^ mice exhibited a two-fold increase in the number of osteoclasts compared to OVX *Osx:cre* mice, and a significant increase in resorption activity when compared to the sham *Osx:cre* mice. It is therefore plausible that ephrinB1, expressed by osteoblasts, interacts with an Eph receptor expressed by monocytes/osteoclast progenitors to inhibit osteoclast formation and activity. While Zhao and colleagues have reported that ephrinB1 and ephrinB2 are expressed by osteoclasts, but lack EphB expression^6^, other studies have reported that low levels of EphB receptors are expressed by murine osteoclasts^42^. However, it has been shown that EphB4 indirectly suppresses osteoclast differentiation by inhibiting RANKL production on osteoblasts^10^. Importantly, the study showed that the direct delivery of EphB4 inhibitor (sEphB4) did not influence osteoclast production, demonstrating that EphB4 was not required for osteoclast formation. Rather administration of sEphB4, during co-culture of osteoblasts and osteoclast precursors was required to inhibit EphB4 on osteoblasts; which subsequently enhanced RANKL production and therefore osteoclast formation^10^. However, these studies have been conducted on mouse primary cells or cell lines.

The present study, sought to determine whether the role of EphB/ephrinB1 signaling during osteoclast differentiation was similar between human samples, and that observed in the ephrinB1 mouse knockout studies. Our observations showed that EphB receptors were expressed by human peripheral blood mononuclear cells and differentiated osteoclasts. The expression of EphB2, the high affinity receptor for ephrinB1, was down-regulated during osteoclast differentiation. Functional studies found that stimulation of human peripheral blood monocytes (osteoclast precursors) with soluble ephrinB1-Fc, inhibited TRAP^+^ multinucleated osteoclast formation, function and associated gene expression, compared to the human-IgG control. To confirm that ephrinB1 mediated its inhibitory function through EphB2 expressing osteoclast precursors, an EphB2-specific blocking peptide was utilised. This peptide functions by competing with the ephrin ligand to bind to the ligand binding domain of EphB2^35^. The addition of the EphB2 blocking peptide reversed the inhibition of osteoclast formation in the presence of ephrinB1-Fc. As previously mentioned, osteoclasts and their precursors also express ephrinB ligands^6^, and it is therefore plausible to suggest that osteoclast precursors may interact with each other to mediate EphB2-ephrinB1 signalling. However, when hPBMNC were cultured under osteoclast conditions in the presence of the EphB2 blocking peptide but in the absence of ephrinB1-Fc, there was no significant difference in the number of TRAP + osteoclasts enumerated when compared to the scramble peptide control (data not shown). This observation is consist with previous studies investigating ephrinB2-EphB4 communication between osteoclasts using the EphB4 inhibitor^10^. Collectively, these observations suggest that the communication between EphB2-ephrinB1 within the osteoclast precursor population is not sufficient to activate EphB2 signalling. Rather, this study proposes that ephrinB1 expressing osteogenic cells directly interact with and activate EphB2 expressing osteoclast precursors to inhibit osteoclast differentiation. The findings confirm that EphB2/ephrinB1 signaling during osteoclast differentiation is conserved between mouse and human. Where the loss of ephrinB1 by osteoblasts in *EfnB1*_OB_^−/−^ mice, enhanced osteoclast function, while the administration of ephrinB1 during osteoclast differentiation of human PBMNC inhibited osteoclast formation and function. Therefore, ephrinB1 appears to act as a negative regulator of osteoclast formation via EphB2 forward signaling. This is consistent with the reported role of EphA4, which negatively regulates osteoclast resorption activity^43^. In contrast, interactions between EphA2/ephrinA2, have been reported to enhance osteoclast differentiation, while suppressing osteoblast differentiation^44^. Therefore, different Eph/ ephrin pairings may provide alternate mechanisms to “fine tune” different phases of bone remodelling during normal bone homeostasis or under pathological conditions.

Collectively, these findings demonstrate the importance of ephrinB1 signalling in maintaining healthy bone, where loss of ephrinB1 in osteoprogenitors not only perturbs osteoblast function, but also affects osteoclast formation. Future studies are required to identify the downstream signaling pathways and mechanism activating osteoclast formation and function in response to EphB2-ephrinB1 signalling between the osteoclastic-osteogenic populations.

## Electronic supplementary material

Supplementary Figures

## Data Availability

All data generated or analysed during this study are included in this published article.
