# Supplementary material for: The osteoprogenitor-specific loss of ephrinB1 results in an osteoporotic phenotype affecting the balance between bone formation and resorption
Source: Sci Rep. 2018 Aug 24;8:12756. doi: 10.1038/s41598-018-31190-2 (PMC6109077; doi:10.1038/s41598-018-31190-2)
Supplement: Supplementary file 1 — Supplementary Figures [file 41598_2018_31190_MOESM1_ESM.pdf]

The osteoprogenitor-specific loss of ephrinB1 results in an osteoporotic phenotype affecting the balance between bone formation and resorption

Agnieszka Arthur[1,2] PhD, Thao M. Nguyen[1,2] PhD, Sharon Paton[1,2,3] BSc, Ana Klisuric[1] BSc Hons, Andrew C.W. Zannettino[2,3] PhD and Stan Gronthos[1,2] PhD.

[1]Mesenchymal Stem Cell Laboratory, Adelaide Medical School, Faculty of Health and Medical Sciences, University of Adelaide, Adelaide 5005, SA, Australia.

[2]South Australian Health and Medical Research Institute, Adelaide 5000, SA, Australia.

[3]Myeloma Research Laboratory, Adelaide Medical School, Faculty of Health and Medical Sciences, University of Adelaide, Adelaide 5005, SA, Australia.

***Corresponding author:** Professor Stan Gronthos

Mesenchymal Stem Cell Laboratory, Adelaide Medical School, Faculty of Health and Medical Sciences, University of Adelaide, Adelaide 5005, SA, Australia.

Email: stan.gronthos@adelaide.edu.au

Running Title: loss of ephrinB1 results in an osteoporotic phenotype

Key words: Osteoporosis, osteoblasts, osteoclasts, molecular pathways – remodelling, Eph/ephrin molecules

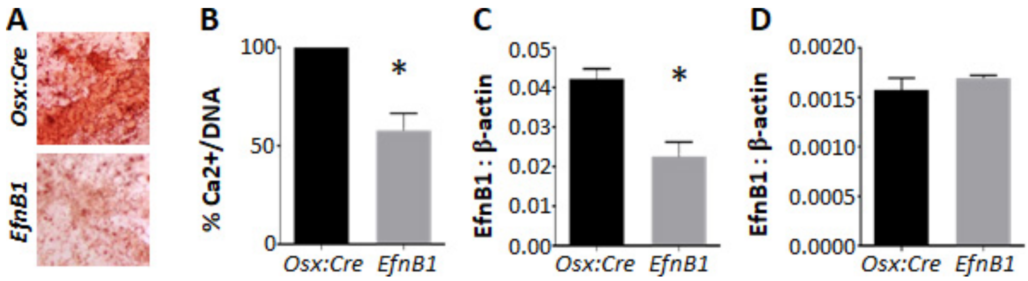

Supplementary Figure 1. Loss of EfnB1 in osteoprogenitors mediated through the osterix promoter influences osteoblast in vitro. (**A-C**) *Osx:Cre* (*Osx*) and *EfnB1*$_{\mathrm{OB}}^{-/-}$ (*EfnB1*) cells were cultured under osteogenic differentiation conditions for 21 days. Cultures were either (**A**) stained with Alizarin Red, (**B**) quantitated for calcium ($Ca2^{+}$) levels and represented as a percentage of $Ca2^{+}$/DNA relative to the *Osx:Cre* control, or (**C**) isolated for PCR analysis of *ephrinB1* gene expression. (**D**) *Osx:Cre and EfnB1*$_{\mathrm{OB}}^{-/-}$ bone marrow cells were cultured under osteoclast conditions. Samples were isolated for PCR analysis of *ephrinB1* gene expression. (All data presented, n = 2-3 mice/strain, * $p<0.05$, Student t-test).

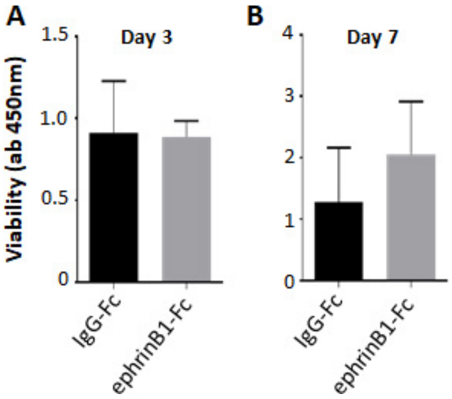

Supplementary Figure 2. Soluble ephrinB1 does not influence osteoclast precursor viability or metabolic function. (**A-B**) Human PBMNC viability and metabolic function was assessed by WST-1 analysis (absorbance 450nm) on (**A**) Day 3 and (**B**) Day 7 following osteoclast differentiation, in the presence of human IgG-Fc or ephrinB1-Fc (n = 2 human donors, Student t-test).
